# Likely uptake of a future a lung cancer screening programme in Hodgkin lymphoma survivors: a questionnaire study

**DOI:** 10.1186/s12890-022-01959-3

**Published:** 2022-04-28

**Authors:** Rachel Broadbent, Christopher J. Armitage, Philip Crosbie, John Radford, Kim Linton

**Affiliations:** 1 Division of Cancer Sciences, School of Medical Sciences, Faculty of Biology, Medicine and Health, University of Manchester, Manchester, UK; 2 NIHR Greater Manchester Patient Safety Translational Research Centre, University of Manchester, Manchester, UK; 3 The Christie NHS Foundation Trust, Manchester, UK; 4 Manchester Cancer Research Centre, Wilmslow Road, Manchester, M20 4QL UK; 5 Division of Infection, Immunity and Respiratory Medicine, University of Manchester, Manchester, M20 4BX UK; 6 Manchester Thoracic Oncology Centre, North West Lung Centre, Manchester University NHS Foundation Trust, Wythenshawe, M23 9LT UK; 7 Manchester Centre for Health Psychology, Division of Psychology and Mental Health, University of Manchester, Manchester, UK; 8 Manchester Academic Health Science Centre, Manchester University NHS Foundation Trust, Manchester, UK

**Keywords:** Hodgkin lymphoma, Lung cancer, Screening, Willingness

## Abstract

**Background:**

Many Hodgkin lymphoma (HL) survivors are at increased risk of subsequent malignant neoplasms (SMN), including lung cancer, due to previous treatment for HL. Lung cancer screening (LCS) detects early-stage lung cancers in ever smokers but HL survivors without a heavy smoking history are ineligible for screening. There is a rationale to develop a targeted LCS. The aim of this study was to investigate levels of willingness to undergo LCS in HL survivors, and to identify the psycho-social factors associated with screening hesitancy.

**Methods:**

A postal questionnaire was sent to 281 HL survivors registered in a long-term follow-up database and at increased risk of SMNs. Demographic, lung cancer risk factors, psycho-social and LCS belief variables were measured. Multivariable logistic regression analysis was performed to determine the factors associated with lung cancer screening hesitancy, defined as those who would ‘probably’ or ‘probably not’ participate.

**Results:**

The response rate to the questionnaire was 58% (n = 165). Participants were more likely to be female, older and living in a less deprived area than non-participants. Uptake (at any time) of breast and bowel cancer screening among those previously invited was 99% and 77% respectively. 159 participants were at excess risk of lung cancer. The following results refer to these 159. Around half perceived themselves to be at greater risk of lung cancer than their peers. Only 6% were eligible for lung cancer screening pilots aimed at ever smokers in the UK. 98% indicated they would probably or definitely participate in LCS were it available. Psycho-social variables associated with LCS hesitancy on multivariable analysis were male gender (OR 5.94 CI 1.64–21.44, *p* < 0.01), living in an area with a high index of multiple deprivation decile (deciles 6–10) (OR 8.22 CI 1.59–42.58, *p* < 0.05) and lower levels of self-efficacy (OR 1.64 CI 1.30–2.08 *p* < 0.01).

**Conclusion:**

HL survivors responding to this survey were willing to participate in a future LCS programme but there was some hesitancy. A future LCS trial for HL survivors should consider the factors associated with screening hesitancy in order to minimise barriers to participation.

**Supplementary Information:**

The online version contains supplementary material available at 10.1186/s12890-022-01959-3.

## Background

Hodgkin lymphoma (HL) is a lymphoid malignancy of clonal B cells predominantly affecting the young and the elderly and accounts for 68% of lymphomas in 15–24 year olds [1, 2]. Whilst over 90% of patients diagnosed under the age of 50 are cured with chemotherapy and/or radiotherapy, 5 year survival rates fall with increasing age at diagnosis [1]. As a consequence of treatment with alkylating agents—specifically mechlorethamine (also known as mustine) and procarbazine—and radiation [3], survivors of HL are at excess risk of developing subsequent malignant neoplasms (SMN), which are the primary cause of death among long-term survivors [4]. The most common SMNs in HL survivors are breast cancer (cumulative incidence (CI) 35-years post-treatment 14.4%) and lung cancer (CI 35-years post-treatment 3.1% in women and 5.2% in men) [5]. However, the SMNs most commonly associated with mortality are gastrointestinal cancers and lung cancer, with an absolute risk of death of 10.4 and 9.4 respectively [6, 7]. In the case of lung cancer, a large case–control study found the relative risks for lung cancer in HL survivors following alkylating agents, radiation to lung ≥ 5 Gy, or both to be 7.2, 4.3 and 7.2 respectively in light or never smokers, increasing in a multiplicative fashion to 20.2, 16.3 and 49.1 respectively in moderate to heavy smokers [3]. Despite these excess risks, the only comprehensive screening programme for the detection of SMNs in the UK is the breast cancer screening programme for women at very high risk of breast cancer (defined by the NHS Breast Screening Programme as a lifetime risk of at least 40% due to a confirmed pathological germline variant or following radiotherapy to breast tissue under age 31 years for the treatment of lymphoma or, rarely, another cancer).) This programme provides for annual breast screening starting 8 years after treatment and continuing until age 70 when the screening frequency reduces to every 3 years [8].

Screening for lung cancer using a low-dose CT scan detects early stage, asymptomatic lung cancers and has been shown to reduce lung cancer mortality in current and former smokers in two large randomised controlled trials [9, 10]. In the United Kingdom (UK), lung cancer screening is being piloted by the National Health Service (NHS) in former or current smokers aged 55–74, who have a 6-year lung cancer risk of ≥ 1.51% according to the PLCOm2012 calculator or a 5-year risk of ≥ 2.5% according to the LLPv2 calculator. The variables entered into these risk calculators are listed in Table 1. Although personal cancer history is included in both calculators, cancer treatments which increase lung cancer risk (thoracic radiotherapy and certain alkylating agents) are not [11].

**Table 1:** Variables entered into the PLCOm2012 and LLPv2 lung cancer risk calculators

	PLCOm2012	LLPv2
Age	✓	✓
Race	✓	✗
Education level	✓	✗
Body mass index	✓	✗
COPD	✓	✗
Pneumonia	✗	✓
Occupational exposure to asbestos	✗	✓
Personal history of cancer	✓	✓
Family history of lung cancer	✓	✓ (in a first degree relative < 60)
Smoking history	✓ (cigarettes per day, duration of smoking, duration of quitting)	✓ (smoking duration only)

Rates of smoking among HL survivors are low [12], and since the lung cancer risk calculators aimed at ever smokers do not take into account the risks associated with prior cancer treatment with radiation and alkylating agents, many HL survivors will not be captured by the lung cancer screening pilots aimed at ever smokers. For this reason, a future lung cancer screening programme for HL survivors must target this population, much like the approach to breast cancer screening.

The positive attitudes of the general public to cancer screening in the UK [13] are reflected in the relatively high levels of uptake for NHS breast, cervical and bowel cancer screening programmes compared to other countries in Europe [14]. However, uptake of lung cancer screening by ever smokers has been suboptimal; the UK-based Lung Screen Uptake Study reported a 53% uptake rate, the highest reported rate among historically low uptake rates for lung cancer screening pilots and trials [15]. That said, uptake of cancer screening is higher among cancer survivors than non-cancer survivors [16, 17] and in a qualitative study in the UK, HL survivors were motivated to participate in a future lung cancer screening programme and reported few barriers to participation [18]. However, it is likely that some of the sociodemographic and psychological barriers to cancer screening in the general public will also apply to HL survivors. This area is worthy of further investigation because uptake of a future targeted lung cancer screening programme could be optimised by interventions designed to minimise known barriers to uptake. In the general population, sociodemographic variables associated with reduced screening participation include older age, male gender and lower socioeconomic status, although the association varies across different screening programmes. Lower levels of education and health literacy—which correlate with lower socioeconomic status—have also been associated with reduced screening participation [19].

Theories such as the Health Belief Model (HBM) have been used to explain variation in screening participation. The HBM constructs of perceived susceptibility, perceived severity, perceived benefits and barriers and self-efficacy have been shown to predict cancer screening uptake [20, 21]. Other factors predictive of non-participation in cancer screening programmes include worse self-rated health [22] and lower levels of dispositional optimism [23], whilst higher levels of cancer worry are both a facilitator and a barrier to participation [24, 25]. Smoking is widely understood by the public as being an important risk factor for lung cancer but current smokers are less likely to participate in lung cancer screening than former smokers [26, 27]. The aim of this study was to use quantitative methods to describe the psychosocial factors associated with hesitancy to participate in a future lung cancer screening programme in HL survivors.

## Methods

### Subjects and setting

Potential participants were identified from a prospective database of ≥ 5 year lymphoma survivors (ADAPT) held at The Christie NHS Foundation Trust. The ADAPT database contains the details of patients treated for high-grade lymphoma (or who were followed-up after completing treatment elsewhere) at The Christie NHS Foundation Trust. The database is prospectively maintained and contains details for patients treated since 1964. Patients are offered entry into the ADAPT GP-led follow-up programme if they remain in remission 5 years after completion of treatment, but they are not discharged from their clinical team. The database contains the names of the chemotherapy regimens received by patients and the anatomical sites which received a dose of radiation.

**Fig. 1:**
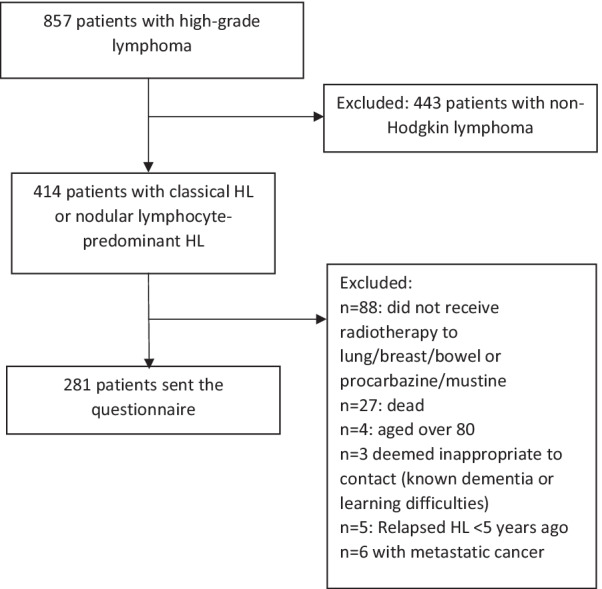
Diagram showing the process of selecting eligible individuals

To identify individuals eligible for this study, patients with classical HL or nodular lymphocyte-predominant HL (n = 414) were identified from the database, which held the records of 857 patients on 18th March 2021. The following patients were then excluded: patients who had died (n = 27), patients aged over 80 (n = 4), patients who had relapsed with HL within the last 5 years (n = 5), patients currently being treated for metastatic cancer at The Christie (n = 6), patients who it was deemed inappropriate to contact due to a diagnosis of dementia or learning difficulties (n = 3), patients who had not received a treatment known to increase their risk of breast cancer (radiation to the breast tissue) [28], bowel cancer (procarbazine or radiation to the bowel) [29] or lung cancer (mustine or procarbazine or radiation to the lung) [3] (n = 88). After applying these criteria to the database, 281 individuals were deemed to be eligible. Figure 1 demonstrates this selection process.

A postal questionnaire and participant information sheet was sent to 281 eligible individuals followed by reminder letters to those who had not returned the questionnaire within 3 weeks. Return of the questionnaire was taken as consent to participate.

### Measures

#### Willingness to undergo lung cancer screening

Participants were asked to rate the strength of their willingness to participate in a future lung cancer screening programme with the question ‘If you were invited to go for a lung cancer screening test, would you go?’ The response options were ‘yes definitely’, ‘yes probably, ‘probably not’ and ‘definitely not’.

#### Lung cancer screening related health beliefs

Lung cancer screening related health beliefs were measured using the Lung Cancer Screening Health Belief Scales (LCSHBS), developed to measure health beliefs impacting lung cancer screening uptake using the HBM framework and psychometrically tested in ever smokers [30]. The LCSHBS comprise of four scales measuring perceived risk of developing lung cancer and perceived benefits, perceived barriers and self-efficacy (an individuals’ belief in their capacity to execute a behaviour) for undergoing lung cancer screening. Although the Extended Health Belief Model includes separate constructs for perceived risk and perceived severity, the LCSHBS do not include a perceived severity scale because cancer is always perceived to be severe. To adapt the LCSHBS for this study population, items relating to cost, lack of a regular healthcare provider and booking a scan appointment were removed and never smokers were instructed not to complete the items in the perceived barriers scale which relate to a personal history of smoking. Prior to completing the scales, participants were provided with a short statement describing a lung cancer screening test.

Items in the perceived risk, perceived benefits and perceived barriers scales were scored using 5-point Likert scales indicating agreement (strongly agree/agree/neither agree nor disagree/disagree/strongly disagree). The self-efficacy scale had a 4-point Likert scale indicating level of confidence (very confident/somewhat confident/slightly confident/not at all confident). The following are examples of items included in the scales: ‘It is likely that I will get lung cancer in the next 5 years’ (perceived risk scale); ‘Having a lung scan would lower my chances of dying from lung cancer’, ‘Having a lung scan would help me plan for the future’ (perceived benefits scale); ‘I might put off a lung scan because no one in my family had lung cancer’, ‘I might put off having a lung scan because I think I am too old to benefit from screening for lung cancer’ (perceived barriers scale; ‘How confident are you that you could find transportation to get to and from the clinic/hospital to have a lung scan?’, ‘How confident are you that you could get a lung scan even if you were anxious about the results?’ (self-efficacy scale).

Cronbach’s alpha was used to estimate internal consistency for each of the LCSHBS subscales and was found to be 0.90 for the 3-item perceived risk scale, 0.84 for 6-item perceived benefits scale, 0.89 for the 7-item self-efficacy scale, 0.94 for the 15-item perceived barrier scale for ever smokers and 0.91 for the 12-item perceived barrier scale for never smokers.

### Demographic factors

Participants’ age, gender and full postcode were extracted from electronic medical records. The questionnaire included questions about ethnicity, current employment status and level of education. Participants’ postcodes were used to calculate area-level socioeconomic deprivation using the Index of Multiple Deprivation (IMD). The IMD combines seven domains of deprivation to produce a rank indicating the relative level of deprivation in a small area [31]. Participants’ IMD ranks were categorised into deciles. The IMD has been used as a measure of socio-economic deprivation in studies examining sociodemographic predictors of cancer screening uptake, including lung cancer screening [15, 26, 32], and researchers have previously categorised IMD ranks into quintiles and tertiles for statistical analysis.

### Other psychosocial and health related factors

Cancer worry was measured using an item adapted from the Cancer Worry Chart [33] which is considered to measure cancer worry severity: ‘*In the last 4 weeks*, how often were you bothered by thoughts or worry about your chances of getting cancer again in the future?’ (response options not at all/slightly/moderately/quite a bit/extremely) [34]. Dispositional optimism was measured using the Revised Life Orientation Test (LOT-R) [35], in which a higher score represents a higher level of dispositional optimism. Self-rated health was measured with a single item taken from the SF-12 Health Survey [36]. Optimistic bias was measured using an existing question relating to developing melanoma [37] adapted for this study: Compared to the average person of your age and sex, how likely is it in your opinion that you will develop [lung] cancer? (response options: much less likely/a bit less likely/about the same/a bit more likely/much more likely/I don’t know). We developed items to measure presence of a close family history of lung cancer (in parents or siblings), prior uptake of breast or bowel cancer screening and prior knowledge of lung cancer as a late effect of HL treatment. To investigate 6-year lung cancer risk values in our participants, demographic and lung cancer risk factor data were entered into the PLCOall2014 (Prostate, Lung, Colorectal, Ovarian) lung cancer risk calculator. The PLCOall2014 calculator is analogous to the PLCOm2012 calculator designed for ever smokers which is currently used to determine eligibility to undergo lung cancer screening in the UK, however PLCOall2014 also calculates 6-year lung cancer risk in never smokers. When it was used to calculate 6-year lung cancer risk in 65,711 never smokers in the PLCO cohort, the maximum risk observed was 1.47% falling below the ≥ 1.51% risk threshold for screening [38].

### Statistical analysis

Descriptive statistics were used to analyse the demographic and clinical characteristics of participants at risk of lung cancer, their knowledge of lung cancer risk, cancer screening behaviours and future lung cancer screening willingness and responses to the LCSHBS.

The demographic characteristics of participants versus non-participants were compared using Chi-squared test for gender and Mann–Whitney U-test for age and Index of Multiple Deprivation (IMD) decile. In relation to the characteristics of participants and non-participants, effect sizes are presented using Cohens *d* values, which have been defined as small (*d* = 0.2), medium (*d* = 0.5), and large (*d* = 0.8) [39]. To identify the psycho-social factors associated with lung cancer screening hesitancy—defined as those responding ‘yes probably’ or ‘probably not’ to the lung cancer screening willingness question—a binary logistic regression analysis was performed. The dependent variable was screening willingness and participants for whom complete data was available for the independent variables were included in the analysis. Independent variables included socio-demographics, psychological variables (cancer worry and LOT-R scale score) and LCSHBS scores. Independent variables were entered into the multivariable logistic regression model regardless of whether they were associated with screening hesitancy on univariate analysis.

For the logistic regression, LCSHBS scoring for perceived risk, perceived benefits and perceived self-efficacy was reversed so that higher scores represented lower risk perception, lower perceived benefits and lower-self-efficacy. Scores for perceived barriers were retained so that higher scores represented higher perceived barriers. This change was made because we hypothesised that higher perceived barriers would increase screening hesitancy, whilst higher perceived risk, benefits and efficacy scores would reduce hesitancy. The following were treated as continuous variables: age, years since treatment, LOT-R score, self-rated health score, cancer worry severity score, perceived risk, benefits barriers and self-efficacy. Likert scale response values were converted to numerical values for the self-rated health and cancer worry severity score measures. The following variables were categorical: gender, IMD decile [categorised as low (deciles 1–5) or high (deciles 6–10)], family history of lung cancer (present or not present), smoking status (never smokers versus current or former smoker). IMD deciles were calculated by postcode using the English IMD 2019 data (276 recipients), Welsh 2019 data (4 recipients) and Scottish 2020 IMD data (1 recipient). A *p* value < 0.05 (two‐tailed) was considered statistically significant for all analyses. Statistical analyses were performed using SPSS 23.0 (IBM, Chicago, IL).

## Results

165/281 questionnaires were returned (58% response rate). The characteristics of participants and non-participants are shown in Table 2. Compared to non-participants, participants were more likely to be female (*p* < 0.01), older (*p* < 0.01) and living in a less deprived area (< 0.05).

**Table 2:** Characteristics of participants and non-participants

	Participants (n = 165)	Non-participants (116)	*p* value	Effect size (cohens d value)
Gender				
Male	69 (42%)	68 (59%)	< 0.01	0.16
Female	96 (58%)	48 (41%)		
Age (median)	55	49	< 0.01	0.54
Index of multiple deprivation (IMD) decile (median)	7	5	< 0.05	0.28

### Participants at excess risk of lung cancer

159 out of 165 participants were at risk of lung cancer due to their treatment for HL (prior receipt of an alkylating agent known to increase lung cancer risk and/or a radiation dose to the lung.) Subsequent data presented in this paper refers to these 159 individuals. The median age was 55, 60% were female, 92% were of white British ethnicity, 38% were current or former smokers and 7% were current smokers. The median number of years since diagnosis and last HL treatment was 24 and 23 years respectively. In terms of treatment for HL, 144 (90.5%) had received radiotherapy, which was most commonly delivered to the mediastinum, and 150 (94%) had received chemotherapy, of whom 62% had received alkylating agents known to increase lung cancer risk [procarbazine or mechlorethamine (also known as mustine)]. The cause of excess lung cancer risk was a combination of an alkylating agent and radiation to the lung in 49%, radiation alone in 41.5% and alkylating agent alone in 9.5%. The demographic and clinical features of the study participants at risk of lung cancer are shown in Table 3.

**Table 3:** Characteristics of participants at excess risk of lung cancer

*Clinical and demographic features of participants at excess risk of lung cancer n = 159*	
Current age: median (range)	55 (29–80)
Gender	Female: 96 (60.3%)
	Male: 64 (39.7%)
Ethnicity	White British: 147 (92%)
	Other^a^: 12 (8%)
Level of education (n = 156)	Education below university level: 86 (54.7%)
	University educated: 57 (37%)
	No educational qualifications: 13 (8.3%)
Employment (n = 158)	Full or part time employed (or in full time education/training): 101 (64%)
	Retired: 40 (25.3%)
	Other: 17 (10.7%)
HL classification	Classical HL: 150 (94%)
	Nodular lymphocyte predominant HL: 9 (6%)
Years since diagnosis: median (range)	24 (6–48)
Time since last treatment: median (range)	23 (6–44)
Sites of radiation (lung and non-lung) n = 144	Mediastinal ± other area: 95 (66%)
	Mantle field ± other area: 28 (19%)
	Other area: 21 (15%)
Chemotherapy regimens^b^ (n = 150)	ChlVPP/EVA only: 46 (31%)
	ABVD only: 43 (29%)
	MVPP only: 19 (13%)
	Multiple chemotherapy regimens: 32 (21%) (of whom 17 underwent stem cell transplant and of whom 28 received procarbazine or mechlorethamine)
	VAPEC-B only: 10 (6%)
Cause of excess lung cancer risk by treatment modality	Radiation to lung and alkylating agent: 78 (49%)
	Radiation to lung only: 66 (41.5%)
	Alkylating agent only: 15 (9.5%)
Smoking history (n = 157)	Never smokers: 96 (62%)
	Former smokers: 49 (31%)
	Current smokers: 12 (7%)
Family history of lung cancer (n = 159)	In parents or siblings: 12 (8%)
	Another family member: 20 (13%)
Self-rated health (n = 157)	Excellent/very good: 46 (29.3%)
	Good/fair: 97 (61.8%)
	Poor/very poor: 14 (8.9%)
Revised Life-Orientation Test scores (possible range 0–24): median (range)	15 (0–23)

^a^Other ethnicities: 2 Indian, 2 Irish, 1 White and Black Caribbean, 1 Mixed (Arab and British), 2 Arab, 1 Bangladesh, 1 African, 1 Caribbean, 1 East African and Asian

^b^*ChlVPP-EVA* chlorambucil, vinblastine, procarbazine, prednisolone, etoposide, vincristine, doxorubicin, *ABVD* doxorubicin, bleomycin, vinblastine, prednisolone), *MVPP* mechlorethamine, vinblastine, procarbazine, prednisolone, *VAPEC-B* doxorubicin, cyclophosphamide, etoposide, vincristine, bleomycin, prednisolone

### Lung cancer knowledge, beliefs and willingness to be screened

31% of participants selected lung cancer as being a late effect of treatment from a list of health conditions. 82/158 (52%) of participants who answered the question about comparative risk of lung cancer believed that their personal risk was higher than the average person of their age and sex, 43 (27.2%) believed they were at equal risk, 8 (5%) at lower risk and 25 (16%) did not know. 52/158 (33%) of participants (32 women, 20 men) had previously been invited to undergo bowel cancer screening, of whom 40 (77%) had taken up the offer at least once (25 women, 15 men). Among female participants, 90/95 (95%) had previously been invited to undergo breast cancer screening, of whom 89 (99%) had taken up the offer at least once. Possible score ranges, median scores, range and interquartile range for the perceived risk, perceived benefits, perceived barriers and self-efficacy scales are shown in Table 4.

**Table 4:** Lung cancer screening health belief scale scores

	Median (range; IQR)
Perceived risk score (possible range 3–15) n = 159	9 (3–15; 3)
Perceived benefits score (possible range 6–30) = 158	24 (11–30; 5)
Perceived barriers score in ever smokers (possible range 15–75) n = 59	23 (15–61; 14)
Perceived barriers score in never smokers (possible range 12–60) n = 94	16 (12–40; 10)
Self-efficacy score (possible range 7–28) n = 157	28 (17–28; 3)

Out of 157 participants who answered the question ‘If you were invited to go for a lung cancer screening test, would you go?’ 127 (81%) responded ‘yes, definitely’, 27 (17%) responded ‘yes, probably’ and 3 (2%) responded ‘probably not’. There were no distinct commonalities among the three participants who indicated that they would probably not attend lung cancer screening compared to those responding yes probably/definitely. The single female responder who would probably not attend lung cancer screening was among the 1% of participants who had not participated in breast cancer screening despite being invited.

PLCOall2014 scores were calculable for 130 participants. The median 6-year lung cancer risk was 0.09% (range < 0.001–8.2%). Thirteen (10%) participants—who were all former or current smokers—met the risk threshold for screening (≥ 1.51%), but when the age bracket for lung cancer screening in the UK (55–74) was applied, just 6% would be eligible for lung cancer screening in the UK through pilots aimed at ever smokers.

A logistic regression analysis was performed to identify factors associated with lung cancer screening hesitancy. 158 participants with complete data for the dependant and independent variables were included in the model. The overall model was statistically significant when compared to the null model (*p* < 0.01), explained 59% of the variation in screening willingness and correctly predicted 90.5% of cases. On univariate analysis, the following factors were associated with screening hesitancy: being male (odds ratio (OR) 2.52, confidence interval (CI) 1.13–5.61) *p* < 0.05), lower perceived benefits (OR 1.29 CI 1.14–1.47 *p* < 0.01), higher perceived barriers (OR 1.09 CI 1.05–1.15 *p* < 0.01) and lower self-efficacy (OR 1.45, CI 1.27–1.65, *p* < 0.01).

On multivariable analysis, the following factors were associated with screening hesitancy: being male (OR 5.94 CI 1.64–21.44, *p* < 0.01), living in an area with a high IMD decile (deciles 6–10) (OR 8.22 CI 1.59–42.58, *p* < 0.05) and lower levels of self-efficacy (OR 1.64 CI 1.30–2.08 *p* < 0.01). The results of the univariable and multivariable analyses are shown in Table 5. For variables with statistical significance, OR, CI and *p* value are in bold.

**Table 5:** Factors associated with lung cancer screening hesitancy (n = 158)

Variable	Univariable			Multivariable		
	Odds ratio	Confidence interval	*p* value	Odds ratio	Confidence interval	*p* value
Male gender	**2.52**	**1.13–5.61**	**< 0.05**	**5.94**	**1.64–21.44**	**< 0.01**
Age	1.01	0.95–1.05	0.41	0.99	0.92–1.06	0.87
Years since treatment	0.98	0.93–1.03	0.44	0.91	0.83–1.00	0.06
LOT-R score	1.04	0.95–1.14	0.31	1.18	0.97–1.43	0.08
Living in an area with a high IMD decile	1.43	0.61–3.37	0.40	**8.22**	**1.59–42.58**	**< 0.05**
No family history of lung cancer	1.23	0.25–5.96	0.78	0.17	0.01–2.20	0.17
Never smoker	1.21	0.53–2.74	0.64	0.80	0.20–3.17	0.76
Cancer worry severity score	0.84	0.59–1.20	0.35	1.01	0.51–1.98	0.97
Self-rated health score	1.11	0.75–1.63	0.59	0.60	0.27–1.31	0.20
Lower perceived risk	1.17	0.98–1.40	0.08	1.03	0.73–1.45	0.82
Lower perceived benefits	**1.29**	**1.14–1.47**	**< 0.01**	1.23	0.98–1.53	0.06
Higher perceived barriers	**1.09**	**1.03–1.15**	**< 0.01**	1.03	0.95–1.12	0.37
Lower self-efficacy	**1.45**	**1.27–1.65**	**< 0.01**	**1.64**	**1.30–2.08**	**< 0.01**

## Discussion

In this questionnaire study, a large majority of long-term HL survivor respondents at risk of lung cancer indicated willingness to undergo lung cancer screening, were the test available. The motivations for lung cancer screening reported by participants in our previous qualitative study [18]—namely perceived benefits and desire for reassurance in a population exhibiting high levels of health anxiety—may explain the high levels of positive lung cancer screening intentions reported in this current study.

Upon registration in the ADAPT programme—usually 5 years following completion of treatment—our standard departmental policy provides a written treatment summary to all patients, including information about an excess risk of lung cancer to HL patients treated with thoracic radiotherapy. Although the vast majority of our participants would have received this information, only 31% recalled and selected lung cancer as being a potential late effect. A larger proportion (52%) of our participants considered themselves to be at greater risk of lung cancer than the average person of the same age and sex. Knowledge of smoking as a lung cancer risk factor [40] and a perceptions by HL survivors of cancer treatments as being toxic [18] could have contributed to these comparative risk perceptions, particularly in participants who were not already aware that lung cancer can be a late effect of treatment. These findings demonstrate a lack of knowledge of personal lung cancer risk among HL survivors and reinforce the need for education about lung cancer risk upon invitation to a future lung cancer screening programme.

We hypothesised that few of our participants at risk of lung cancer would be eligible for screening through programmes aimed at ever smokers. Although the demographic characteristics of our participants do not fully reflect the HL survivor population overall (being older and female was associated with participation in the study), we found that just 10% met the≥ 1.51% 6-year lung cancer risk threshold for screening, falling to 6% when the age eligibility criteria for lung cancer screening for ever smokers in the UK were applied. This finding supports our hypothesis and our view that a targeted lung cancer screening programme for HL survivors should be developed. Survivorship care varies widely in the UK and many patients are discharged 2–5 years after achieving remission. Retrospectively identifying HL survivors at risk of lung cancer who have been discharged from their treating centres and who are eligible for lung cancer screening is likely to be time consuming and will require a significant effort from treating centres and potentially collaboration with primary care. A number of approaches have been used to identify and recruit ever smokers to lung cancer screening pilots and trials in the UK, including advertising and using electronic primary-care records, and a future targeted lung cancer screening programme for HL survivors may draw on the relative success of these approaches [41].

The demographic variables associated with screening hesitancy were being male and living in a less deprived area. The impact of gender may be explained by the very high levels of breast cancer screening uptake among female participants. Although cervical screening uptake was not investigated in this survey, it is likely that many of the female participants would also have experience of cervical screening. On the other hand, few male participants had been invited or participated in bowel cancer screening, potentially increasing hesitancy due to reduced levels of awareness around cancer screening and risk. Furthermore, our prior qualitative research found that women viewed breast cancer screening as a norm and their awareness of an excess risk of breast cancer aided their understanding of lung cancer risk [18], perspectives which could have increased their willingness to undergo lung cancer screening. The association between living in a less deprived area and screening hesitancy in this study contrasts with the literature showing that a lower socioeconomic status is associated with lower cancer screening uptake [19]. This discrepancy may be due to this study investigating willingness to participate in a hypothetical screening scenario as opposed to actual lung cancer screening uptake. In reality, people living in more deprived areas are likely to experience greater barriers to participation than those in more affluent areas, such as the ability to take time off work and to travel to a screening appointment.

Overall, our participants exhibited high perceived benefits scores, high self-efficacy scores and low perceived barriers scores. We are not able to compare the scores of our participants with those of other groups firstly because we adapted the LCSHBS for our population and secondly because there is a lack of published studies that have used the scales in their intended population of ever smokers. In our study, the only health belief model construct predictive of screening hesitancy on multivariable analysis was self-efficacy. Self-efficacy is widely considered to be an important predictor of behaviour and is incorporated into numerous theoretical models. The question items relating to self-efficacy used in our study related to finding time to attend, transportation and ability to cope with anxiety about the results and uncertainty about the procedure. Our participants have prior experience of navigating the healthcare system—experience which is likely to be ongoing for many due to the late effects of treatment—of undergoing scans and dealing with the associated anxiety. This prior experience and the fact that health is a priority for this group [12, 18] may explain the high levels of self-efficacy in our participants.

A meta-analysis of health belief model variables in predicting behaviour found that outcome expectancies—perceived benefits and barriers—were the strongest predictors of behaviour [21]. However, neither perceived benefits nor perceived barriers were associated with screening hesitancy in the multivariable analysis in our study. It is possible that outcome expectancies predict intention to decline lung cancer screening by HL survivors, but as there were very few participants who indicated they would decline screening, we could not perform this analysis. With regards to perceived risk, our findings are supported by the aforementioned meta-analysis which did not identify a correlation between susceptibility (perceived risk) and preventative behaviours.

### Strengths and limitations

This study is the first to use quantitative methodology grounded in behavioural theory to explore the psycho-social factors predictive of willingness to undergo lung cancer screening among HL survivors. The study complements and supports our previous qualitative work on this topic and provides further evidence of high levels of willingness among this group to undergo lung cancer screening in the future. The resulting knowledge regarding the psycho-social factors which impact screening hesitancy could inform the design of a future lung cancer screening programme and its’ associated informational materials, with the aim of optimising uptake rates. However, this must be balanced against the need to provide invitees with information about both the potential harms and benefits of screening in order to facilitate informed decision making [42].

The extent to which the findings of this study can be applied to a national HL survivor population is limited by the characteristics of our participants who were registered in a long-term follow-up programme (most HL survivors who are in remission are discharged between 2 and 5 years after completion of treatment) and more likely to be female, older and living in a less deprived area than non-participants. In addition, a large majority of participants were of white British ethnicity and just over a third were university educated. Whilst the impact of gender and age on cancer screening participation rates is not always clear cut, a lower socioeconomic status (which correlates with lower levels of education and higher levels of smoking) has consistently been demonstrated to be a barrier to uptake [19] and people of non-white ethnicity face specific barriers to screening participation [43]. Therefore, the high levels of willingness to undergo lung cancer screening in this study may not reflect the entire HL survivor population, who would be expected to mirror the general population in terms of socioeconomic status and ethnicity. It is also possible that a greater proportion of non-participants to our study would decline lung cancer screening, compared to the participants. If this were the case, our study would have overestimated levels of willingness to undergo lung cancer screening. Although current smokers were poorly represented among our participants, the rates of current smoking in this study (7%), mirror the findings of another study [12], so it may be that rates of current smoking among HL survivors among our participants truly reflect those in the HL survivor population.

## Conclusions

In this study we have identified the psycho-social factors associated with lung cancer screening hesitancy in HL survivors asked to consider a hypothetical lung cancer screening scenario and identified high levels of willingness to participate were lung cancer screening to become available. This study suggests that participation rates in lung cancer screening by HL survivors could be higher than in ever smokers and may exceed breast, cervical and bowel cancer screening uptake by the general population. Lung cancer screening is not routinely available for HL survivors and a trial of screening in this population is required to test lung cancer screening methodology established in ever smokers. Within such a trial, there would be value in exploring motivations and barriers to participation in a real-world setting. Further issues in this area worthy of exploration include developing lung cancer screening informational materials for HL survivors since current materials are aimed towards ever smokers and are not appropriate for use in this group. Developing a lung cancer risk calculator for this population is another important consideration to optimise selection criteria for lung cancer screening.

## Supplementary Information

**Additional file 1**. Study questionnaire.

## Data Availability

All data generated or analysed during this study are included in this published article [and its Additional file 1].

## Abbreviations

HL: Hodgkin lymphoma
SMN: Subsequent malignant neoplasm
UK: United Kingdom
CI: Cumulative incidence
CT: Computed tomography
NHS: National health service
HBM: Health belief model
LCSHBS: Lung cancer screening health belief scales
PLCO: Prostate lung colorectal ovarian
IMD: Index of multiple deprivation
OR: Odds ratio
CI: Confidence interval
