# Supplementary material for: Likely uptake of a future a lung cancer screening programme in Hodgkin lymphoma survivors: a questionnaire study
Source: BMC Pulm Med. 2022 Apr 28;22:165. doi: 10.1186/s12890-022-01959-3 (PMC9052526; doi:10.1186/s12890-022-01959-3)
Supplement: Supplementary file 1 — Additional file 1. Study questionnaire. [file 12890_2022_1959_MOESM1_ESM.docx]

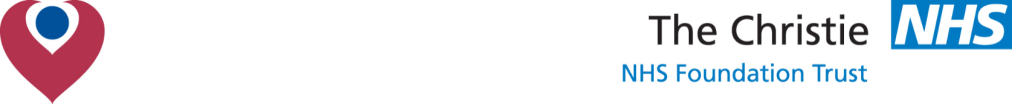
6

A survey exploring health and screening tests in people treated for Hodgkin lymphoma

Study ID:

**Thank you for taking part in this study**.

The study asks for your views on attending various cancer screening tests. It also covers topics such as your health and your lifestyle. It also asks for some information about you.

**How to fill in this questionnaire**

- Please read the instructions and questions carefully.
- To answer the questions, tick the box , write on the dotted line or in the box provided
- Fill in the answer which best describes how you feel.
- Please try to answer all the questions. If you do not wish to answer a question, please leave it blank.
- Do not spend too long on each question – the first answer which comes to you is probably the best one.
- There are no right or wrong answers. If you are unsure about how to answer a question please put the best answer you can.
- You may wish to take breaks while completing the questionnaire
- The information you provide will remain **strictly confidential.**
- Please return your questionnaire for free in the envelope provided
- If you wish to complete the questionnaire online you can do this by emailing [(redacted)](mailto:rachel.broadbent1@nhs.net) and we will send you a link

Part 1: About you and your health

These questions help us to know more about you and your health and help us to better understand the results of this survey. You may have been asked about these issues before in other surveys, but please respond to all the questions if you can.

1) How would you describe yourself? Choose ONE section from A to E then tick ONE box which best describes your ethnic group or background

A) White
 English/Welsh/Scottish/Northern Irish/ British
 Irish
 Gypsy or Irish traveller
 Any other white background please specify:

…………………………………………………………………………………………………………………………………………………………………………………….

B) Black / African / Caribbean / Black British

 African
 Caribbean
 Any other Black / African / Caribbean background please specify:

……………………………………………………………………………………………………………………………………………………………………………………

C) Asian / Asian British

 Indian
 Pakistani
 Bangladesh
 Chinese
 Any other Asian background please specify:

………………………………………………………………………………………………………………………………………………………………………………

D) Mixed / multiple ethnic groups

 White and Black Caribbean
 White and Black African
 White and Asian
 Any other mixed / multiple ethnic background please specify:

…………………………………………………………………………………………………………………………………………………………….

E) Other ethnic group

 Arab

 Any other ethnic background please specify:

..................................................................................................................................................................

2) Which of the following best describes your education? Tick one box

- I left school with no qualifications
- I have GCSEs (previously CSEs) or equivalent
- I have A levels or O-levels
- I did some further education (beyond college) but not a degree
- I have an undergraduate university degree
- I have a postgraduate degree
- Prefer not to say

3) Which of the following best describes your current employment? Tick one box.

Employed full-time

- Employed part-time
- Self-employed
- On sick-leave
- Looking after home or family
- Voluntary work
- Disabled or long-term sick
- Unemployed
- Retired
- In full-time education/training
- In part-time education/training

Other please specify: ……………………………………………………………………………………………

4) For someone your age, how would you rate your health overall? : Tick one box.

- Excellent
- Very good
- Good
- Fair
- Poor
- Very poor

5) Have you ever been diagnosed with COPD or emphysema or bronchitis?

- Yes
- No

6) Which of the following best describes you?

- I have **never smoked** (go to question 9)
- I am an **ex-smoker**

If you are an ex-smokers, how many years ago did you quit smoking? ………………… years

- I **currently smoke**

7) How many years have you/did you smoke for? (Include cigarettes, pipes and cigars).

….................................... years

8) How many cigarettes did/do you smoke on an average day? …………………………

9) a) What is your height?................ cm OR …….. feet …….. inches

b) What is your weight? ……….. kg OR ……. Stones………..pounds

10) *In the last 4 weeks*, how often were you bothered by thoughts or worry about your chances of getting cancer again in the future? Tick one box.

- Not at all
- Slightly
- Moderately
- Quite a bit
- Extremely

11) Please read the statements below carefully and rate how much you agree or disagree with the statement by ticking a box to the right.

| In uncertain times I usually expect the best | Strongly agree | Agree | Neither agree nor disagree | Disagree | Strongly disagree |
| --- | --- | --- | --- | --- | --- |
| It’s easy for me to relax | Strongly agree | Agree | Neither agree nor disagree | Disagree | Strongly disagree |
| If something can go wrong for me, it will | Strongly agree | Agree | Neither agree nor disagree | Disagree | Strongly disagree |
| I’m always optimistic about my future | Strongly agree | Agree | Neither agree nor disagree | Disagree | Strongly disagree |
| I enjoy my friends a lot | Strongly agree | Agree | Neither agree nor disagree | Disagree | Strongly disagree |
| It’s important for me to keep busy | Strongly agree | Agree | Neither agree nor disagree | Disagree | Strongly disagree |
| I hardly ever expect things to go my way | Strongly agree | Agree | Neither agree nor disagree | Disagree | Strongly disagree |
| I don’t get upset too easily | Strongly agree | Agree | Neither agree nor disagree | Disagree | Strongly disagree |
| I rarely count on good things happening to me | Strongly agree | Agree | Neither agree nor disagree | Disagree | Strongly disagree |
| Overall, I expect more good things to happen to me than bad | Strongly agree | Agree | Neither agree nor disagree | Disagree | Strongly disagree |
|  |  |  |  |  |  |
|  |  |  |  |  |  |

Health problems after treatment for Hodgkin lymphoma

12) Cancer treatments can cause health problems later in life. Below is a list of health problems. Please tell us which ones you think **can** be caused by treatment for Hodgkin lymphoma based on your personal knowledge by ticking the box next to the problem.

- High blood pressure
- Breast cancer
- Problems with vision
- Bowel (colorectal) cancer
- Leukaemia
- Weak heart muscles
- Heart attacks
- An underactive thyroid
- Difficulties getting pregnant
- Diabetes
- Lung cancer
- Eczema
- An overactive thyroid
- Asthma or bronchitis
- Arthritis
- Early menopause in women
- Low testosterone levels in men
- Problems with hearing

**Going for cancer screening tests**

The NHS runs cancer screening programs for breast cancer, bowel cancer and cervical cancer. In the future there may be a lung cancer screening program for people at risk.

We would like to know how you feel about your risk of developing cancer in the future and your views on going for cancer screening test for breast, bowel and lung cancer.

| 13) Compared to the average person of your age and sex, how likely is it in your opinion that you will develop the following types of cancer? Circle your answer. | | | | | | |
| --- | --- | --- | --- | --- | --- | --- |
| 1. Breast cancer (women only) | Much less likely | A bit less likely | About the same | A bit more likely | Much more likely | I don’t know |
| 1. Bowel cancer   (men and women) | Much less likely | A bit less likely | About the same | A bit more likely | Much more likely | I don’t know |
| 1. Lung cancer (men and women) | Much less likely | A bit less likely | About the same | A bit more likely | Much more likely | I don’t know |

14) Have you ever *been invited* to do a **bowel cancer** screening home test kit?

- Yes
- No

If the answer was yes, have you *ever had* a **bowel cancer** screening test?

- Yes
- No

15) Women only: Have you ever *been invited* to go for a **breast cancer** screening test (mammogram or MRI scan)?

- Yes
- No

If the answer was yes, have you *ever had* a **breast cancer** screening test (mammogram or MRI scan)?

- Yes
- No

16) A lung cancer screening programme may become available on the NHS in the future. The test used for lung cancer screening is a CT scan of the chest. The scan takes 20 seconds and does not require an injection. Please respond to the following statements about having a lung cancer screening test. Circle your answer.

| It is likely that I will get lung cancer sometime in my lifetime | Strongly Agree | Agree | Neither agree not disagree | Disagree | Strongly disagree |
| --- | --- | --- | --- | --- | --- |
| It is likely that I will get lung cancer in the next ten years | Strongly Agree | Agree | Neither agree not disagree | Disagree | Strongly disagree |
| It is likely that I will get lung cancer in the next five years | Strongly Agree | Agree | Neither agree not disagree | Disagree | Strongly disagree |
| Having a lung scan would help find lung cancer early | Strongly Agree | Agree | Neither agree not disagree | Disagree | Strongly disagree |
| Having a lung scan would lower my chances of dying from lung cancer | Strongly Agree | Agree | Neither agree not disagree | Disagree | Strongly disagree |
| Having a lung scan would help me not worry as much about lung cancer | Strongly Agree | Agree | Neither agree not disagree | Disagree | Strongly disagree |
| Having a lung scan would help me plan for the future | Strongly Agree | Agree | Neither agree not disagree | Disagree | Strongly disagree |
| Having a lung scan would help my family not worry as much | Strongly Agree | Agree | Neither agree not disagree | Disagree | Strongly disagree |
| Having a lung scan would give me peace of mind | Strongly Agree | Agree | Neither agree not disagree | Disagree | Strongly disagree |
| I might put off having a lung scan because I worry about finding something wrong | Strongly Agree | Agree | Neither agree not disagree | Disagree | Strongly disagree |
| I might put off having a lung scan because I don’t have the time | Strongly Agree | Agree | Neither agree not disagree | Disagree | Strongly disagree |
| I might put off a lung scan because no one in my family had lung cancer | Strongly Agree | Agree | Neither agree not disagree | Disagree | Strongly disagree |
| I might put off having a lung scan because I don’t have any lung problems or symptoms | Strongly Agree | Agree | Neither agree not disagree | Disagree | Strongly disagree |
| I might put off having a lung scan because transportation would be a problem | Strongly Agree | Agree | Neither agree not disagree | Disagree | Strongly disagree |
| I might put off having a lung scan because I am afraid the lung scan will damage my lungs | Strongly Agree | Agree | Neither agree not disagree | Disagree | Strongly disagree |
| I might put off having a lung scan because I have had a bad experience with a hospital or healthcare provider | Strongly Agree | Agree | Neither agree not disagree | Disagree | Strongly disagree |
| I might put off having a lung scan because I don’t know enough about the test | Strongly Agree | Agree | Neither agree not disagree | Disagree | Strongly disagree |
| I might put off having a lung scan because I think I am too old to benefit from screening for lung cancer | Strongly Agree | Agree | Neither agree not disagree | Disagree | Strongly disagree |
| I might put off having a lung scan because I would rather **not** know if I have any lung problems | Strongly Agree | Agree | Neither agree not disagree | Disagree | Strongly disagree |
| I might put off having a lung scan because it is not worth the effort | Strongly Agree | Agree | Neither agree not disagree | Disagree | Strongly disagree |
| I might put off having a lung scan because I do not trust the healthcare system | Strongly Agree | Agree | Neither agree not disagree | Disagree | Strongly disagree |

**Only answer the next 3 statements if you have ever smoked tobacco:**

| I might be put off having a lung scan because I currently smoke or used to smoke | Strongly Agree | Agree | Neither agree not disagree | Disagree | Strongly disagree |
| --- | --- | --- | --- | --- | --- |
| I might be put off having a lung scan because I feel like a social outcast for smoking | Strongly Agree | Agree | Neither agree not disagree | Disagree | Strongly disagree |
| I might be put off having a lung scan because I worry about being blamed for having smoked | Strongly Agree | Agree | Neither agree not disagree | Disagree | Strongly disagree |

Please read the statements below carefully and rate your confidence level by ticking a box to the right

| How confident are you that you could find the time to have a lung scan? | Very confident | Somewhat confident | Slightly confident | Not at all confident |
| --- | --- | --- | --- | --- |
| How confident are you that you could find transportation to get to and from the clinic/hospital to have a lung scan? | Very confident | Somewhat confident | Slightly confident | Not at all confident |
| How confident are you that you could get enough information about having a lung scan? | Very confident | Somewhat confident | Slightly confident | Not at all confident |
| How confident are you that you could get a lung scan even if you were worried about the results? | Very confident | Somewhat confident | Slightly confident | Not at all confident |
| How confident are you that you could get a lung scan even if you didn’t know what to expect about the procedure? | Very confident | Somewhat confident | Slightly confident | Not at all confident |
| How confident are you that you could get a lung scan even if you were anxious **about the process**? | Very confident | Somewhat confident | Slightly confident | Not at all confident |
| How confident are you that you could get a lung scan even if you were **anxious about the results**? | Very confident | Somewhat confident | Slightly confident | Not at all confident |

17) Has anyone you know been diagnosed with lung cancer?

- Yes, one of my parents or siblings
- Yes, another family member
- Yes, someone I know but am not related to
- No

18) If you were invited to go for a lung cancer screening test, would you go? Tick one box.

- Yes definitely
- Yes probably
- Probably not
- Definitely not

In the UK the NHS runs a bowel cancer screening programme for men and women aged 60 to 74. The test used in the screening programme is a kit you use at home to collect a small sample of poo, known as the FIT kit.

19) Please answer the following statements about the bowel cancer screening home test kit. Circle your answer.

| It is extremely likely that I will get colorectal cancer in my lifetime | Strongly agree | Agree | Neither agree nor disagree | Disagree | Strongly disagree |
| --- | --- | --- | --- | --- | --- |
| The bowel screening test can find bowel cancer early | Strongly agree | Agree | Neither agree nor disagree | Disagree | Strongly disagree |
| When bowel cancer is found early it can be cured | Strongly agree | Agree | Neither agree nor disagree | Disagree | Strongly disagree |
| Regular bowel screening helps you live longer | Strongly agree | Agree | Neither agree nor disagree | Disagree | Strongly disagree |
| Regular bowel screening helps you to worry less | Strongly agree | Agree | Neither agree nor disagree | Disagree | Strongly disagree |
| Bowel screening gives you peace of mind | Strongly agree | Agree | Neither agree nor disagree | Disagree | Strongly disagree |
| Bowel screening gives you a sense of control over your health | Strongly agree | Agree | Neither agree nor disagree | Disagree | Strongly disagree |
| I might be put off bowel screening because it is embarrassing | Strongly agree | Agree | Neither agree nor disagree | Disagree | Strongly disagree |
| I might be put off bowel screening because it is uncomfortable | Strongly agree | Agree | Neither agree nor disagree | Disagree | Strongly disagree |
| I might be put off bowel screening because it is inconvenient | Strongly agree | Agree | Neither agree nor disagree | Disagree | Strongly disagree |
| I might be put off bowel screening because I don’t want to know if I have bowel cancer | Strongly agree | Agree | Neither agree nor disagree | Disagree | Strongly disagree |
| I might be put off bowel screening because it is a cause for worry | Strongly agree | Agree | Neither agree nor disagree | Disagree | Strongly disagree |
| I am confident that I could manage to do the bowel screening home test kit | Strongly agree | Agree | Neither agree nor disagree | Disagree | Strongly disagree |

20) Has your doctor ever recommended that you should do the bowel screening test when you are invited?

- Yes
- No
- Unsure

21) Has anyone you know been diagnosed with bowel cancer?

- Yes, one of my parents or siblings
- Yes, another family member
- Yes, someone I know but am not related to
- No

22) When you are next invited to do a bowel screening test, will you do it?

- Yes definitely
- Yes probably
- Probably not
- Definitely not

**Only women should answer this next section. For men this is the end of the survey – go to page 18.**

In the UK women aged 50-71 are invited to have a breast cancer screening test (a mammogram).

Women who had radiotherapy to the chest area before the age of 36 are invited to have a breast cancer screening test (either a mammogram or MRI depending on their current age) earlier than the general population.

23) Please answer the following statements about breast cancer screening. Circle your answer.

| It is likely that I will get breast cancer | Strongly agree | Agree | Neither agree nor disagree | Disagree | Strongly disagree |
| --- | --- | --- | --- | --- | --- |
| My chances of getting breast cancer in the next few years are great | Strongly agree | Agree | Neither agree nor disagree | Disagree | Strongly disagree |
| I feel I will get breast cancer sometime during my life | Strongly agree | Agree | Neither agree nor disagree | Disagree | Strongly disagree |
| If I get a breast screening test and nothing is found, I do not worry as much about breast cancer | Strongly agree | Agree | Neither agree nor disagree | Disagree | Strongly disagree |
| Having a breast screening test will help me find breast lumps early | Strongly agree | Agree | Neither agree nor disagree | Disagree | Strongly disagree |
| If a lump is found on my breast screening test, my treatment may not be as bad | Strongly agree | Agree | Neither agree nor disagree | Disagree | Strongly disagree |
| Having a breast screening test is the best way for me to find a very small lump | Strongly agree | Agree | Neither agree nor disagree | Disagree | Strongly disagree |
| Having a breast screening test will decrease my chance of dying from breast cancer | Strongly agree | Agree | Neither agree nor disagree | Disagree | Strongly disagree |
| I might be put off a breast screening test because I might find out something is wrong | Strongly agree | Agree | Neither agree nor disagree | Disagree | Strongly disagree |
| I might be put off a breast screening test because I don’t understand what will be done | Strongly agree | Agree | Neither agree nor disagree | Disagree | Strongly disagree |
| I might be put off a breast screening test because it is embarrassing | Strongly agree | Agree | Neither agree nor disagree | Disagree | Strongly disagree |
| I might be put off a breast screening test because it takes too much time | Strongly agree | Agree | Neither agree nor disagree | Disagree | Strongly disagree |
| I might be put off a breast screening test because it is too painful | Strongly agree | Agree | Neither agree nor disagree | Disagree | Strongly disagree |
| I might be put off a breast screening test because the people who do it are rude to women | Strongly agree | Agree | Neither agree nor disagree | Disagree | Strongly disagree |
| I might be put off a breast screening test because it exposes me to unnecessary radiation | Strongly agree | Agree | Neither agree nor disagree | Disagree | Strongly disagree |
| I might be put off a breast screening test because I have other problems that are more important | Strongly agree | Agree | Neither agree nor disagree | Disagree | Strongly disagree |
| I might be put off a breast screening test because I am too old to benefit | Strongly agree | Agree | Neither agree nor disagree | Disagree | Strongly disagree |
| I am confident that I can go for a breast screening test when invited | Strongly agree | Agree | Neither agree nor disagree | Disagree | Strongly disagree |

Has your doctor ever recommended that you should go for the breast screening test when you are invited?

- Yes
- No
- Unsure

Has anyone you know been diagnosed with breast cancer?

- Yes, one of my parents or siblings
- Yes, another family member
- Yes, someone I know but am not related to
- No

When you are next invited to go for a breast screening test, will you have it?

- Yes definitely
- Yes probably
- Probably not
- Definitely not

**End of survey**

**What to do if you want more information or have concerns**:

If you have any concerns, or would like more information on the late effects of cancer treatment, you can:

- Speak to a lymphoma clinical nurse specialist at The Christie:  (telephone number)
- Seek advice and support from Lymphoma Action- they are aware of this study
- via their website [http://lymphoma-action.org.uk](http://lymphoma-action.org.uk/)
- or their free helpline 0808 808 5555 Monday to Friday 10am-3pm
- a live chat option is available via the ‘Contact Us’ section of the website

If you would like to discuss any aspect of the questionnaire survey you can contact our research team:

By email:

By phone:

By post:

To obtain a summary of the results of this survey, contact Dr Rachel Broadbent using any of the contact methods above.

**Please post your survey back to us using the envelope we have provided.**

Thank you for your participation.
